# The development of an intervention to manage pain in people with late-stage osteoarthritis

**DOI:** 10.4102/sajp.v72i1.311

**Published:** 2016-06-29

**Authors:** Tina Kruger-Jakins, Melissa Saw, Naila Edries, Romy Parker

**Affiliations:** 1 Musculoskeletal Health, ICAS South Africa, South Africa; 2 Physiotherapy Department, Tygerberg Hospital, South Africa; 3 Department of Health & Rehab Sciences, Faculty of Health Sciences, University of Cape Town, South Africa

## Abstract

**Background:**

Osteoarthritis (OA) is one of the most common musculoskeletal conditions worldwide, affecting the functional abilities of millions of people. Arthroplasty is recommended as a successful treatment option for late-stage OA. However, in South Africa there are extensive waiting lists for OA-related arthroplasty in government hospitals. This has negative consequences for patients having to cope for long periods of time with chronic pain and its impact. Alternative treatment methods in the form of physiotherapy-led exercise and education programmes focusing on pain, disability, self-efficacy, physical function and health-related quality of life have had good impact in populations elsewhere.

**Objectives:**

To develop an exercise and education intervention based on the current literature and by doing a field survey in a South African population.

**Results:**

A combined educational approach, with a strong focus on the physical aspects of exercise in particular, was adopted for the intervention in order to improve function and manage the disability associated with OA.

**Conclusion:**

This paper reports on the process and development of an intervention for use in South Africans with late-stage OA awaiting arthroplasty.

## Introduction

Worldwide, the prevalence of musculoskeletal conditions is high (Brooks 2006; Lidgren 2003; World Health Organization 2003). One of the most common musculoskeletal conditions is osteoarthritis (OA), which is accompanied by a range of symptoms including pain and loss of function. OA is considered one of the most common causes of severe long-term pain and disability (Brooks 2002; Helmick *et al*. 2008). Disability is associated with lack of function, which in turn has detrimental consequences for the individual’s activities of daily living and thereby health-related quality of life (Alviar *et al*. 2012; Jelsma *et al*. 2004). In 2003, Woolf and Pfleger reported that the worldwide incidence of OA was on the increase, affecting both men and women, with women being affected at a higher rate than men The trend has continued throughout the last 10 years (Litwic *et al*. 2013; Zhang & Jordan 2010).

In South Africa, the prevalence of OA is similarly high (Usenbo *et al*. 2015). The added complications of low socio-economic demographics, limited access to healthcare, as well as a high prevalence of other chronic conditions, such as HIV straining healthcare resources, makes management of this non-communicable chronic condition challenging (Brooks 2006; Lidgren 2003; World Health Organization 2003). Despite pain being identified as a problem in people living with late-stage OA, the non-pharmacological treatment options such as education, exercise, and surgery to manage the burden of pain in these individuals are limited (Lorig, Ritter & Plant 2005a).

A growing evidence base indicates that intervention programmes for chronic conditions such as OA need to be bio-psychosocial in nature (Lorig *et al*. 2005a, 2005b; Swendeman, Ingram & Rotheram-Bonus 2009). These interventions are proving successful in the management of many different chronic disorders including OA (Hurley *et al*. 2007a; Lorig *et al*. 2005a). However, evidence to support the application of bio-psychosocial treatment interventions in South Africa is lacking. A key component of these bio-psychosocial interventions is health education aimed at enabling patients with chronic disorders to achieve behaviour changes advantageous to health through increasing knowledge and self-efficacy (Hurley *et al*. 2007; Lamb, Toye & Barker 2008).

Evidence suggests several benefits to combining bio-psychosocial education with exercise (Hurley, Mitchell & Walsh 2003; Lamb, *et al*. 2008). The Enabling Self-management and Coping with Arthritic Pain using Exercise (ESCAPE) knee pain programme for knee OA (Hurley *et al*. 2007) is a bio-psychosocial intervention enabling self-management and coping with arthritic knee pain through education and exercise. Such rehabilitation programmes are designed to improve function by integrating exercise, education and self-management strategies to dispel inappropriate health beliefs, alter behaviour and encourage regular physical activity (Hurley *et al*. 2012; Jessep *et al*. 2009). The benefits of the ESCAPE programme include improved function, pain, self-efficacy and health beliefs with large effect sizes. The ESCAPE knee pain programme appears to be an effective and sustainable treatment option having been tested in several robust studies in the United Kingdom (Hurley *et al*. 2012).

Further evidence to support the health and clinical benefits of a bio-psychosocial intervention is provided by the TOAP (Taipei Osteoarthritis Program) (Wu *et al*. 2011). A programme developed and tested in Taipei city districts. Similar to the ESCAPE knee programme, the TOAP focuses on self-efficacy and behaviour changes enabling self-management. The programme is delivered in small groups and facilitated by a trained physiotherapist. TOAP includes patient education, exercise and participation in discussions aiming to assist people to maintain a healthy lifestyle, seek support, solve problems and make action plans (Wu *et al*. 2011), elements which resonate throughout the literature and therefore important to consider in the development of an intervention.

There is a strong rationale for developing and implementing bio-psychosocial interventions for patients with OA. Such a multidimensional model of treatment aligns with the International Classification of Function, Disability and Health (ICF) guidelines (Bagraith & Strong 2013) for including not only the individual but also the surrounding environment combining the whole functional and able individual. In addition to the personal benefits, intervention programmes have been shown to be cost effective, with fewer visits to healthcare facilities (Hurley *et al*. 2007b, 2012). Furthermore, for people eligible for arthroplasty, there is evidence that bio-psychosocial intervention programmes may improve surgical outcomes (Ayers, Franklin & Ring 2013)

Conclusively, because of the strong evidence available internationally with regards to the benefits and safety of self-management focused interventions for people living with OA, the aim of this study was to develop an intervention to manage pain in people living with OA awaiting arthroplasty in South Africa. The majority of South Africans are dependent on public healthcare, and waiting times for elective surgery in this sector is knowingly much longer than in the private sector, often extending into year-long waiting periods (South African Government 2014).

## Method

In order to develop a feasible evidence-based bio-psychosocial intervention to manage pain in people with late-stage OA in South Africa, a three-step approach was adopted. The first step was to draw specific evidence on the management of late-stage OA primarily based on international evidence-based guidelines (Osteoarthritis Research Society International [OARSI] and NICE Guidelines) and their recommendations for the management of OA (McAlindon *et al*. 2014; O’Mahony & Oliver 2008). This was followed by conducting a narrative review of intervention programmes already in use in South Africa for various chronic pain conditions in order to identify components appropriate for pain in OA relevant to the population. The search for such intervention programmes was conducted through PubMed and Elton B. Stephens Company (EBSCO) for all publications up to and including 2013 using the following search terms: *musculoskeletal conditions, arthritis, osteoarthritis, chronic pain, health related quality of life, disability, function, self-efficacy, South Africa, ICF, epidemiology, intervention programs, self-management programs*. In addition, chronic pain management programmes in use at public health institutions were reviewed (Groote Schuur Hospital 2012; Helen Joseph Hospital 2012). Of specific interest were programmes that included both educational and exercise components. Intervention programmes combining both elements show better outcomes than educational programmes alone as they improve functional as well as self-efficacy outcomes (Brady *et al*. 2003; Jensen *et al*. 2013). This was followed by the final step of a field search to locate appropriate settings for testing the intervention.

Evidence was drawn from the literature on intervention programmes already in place to guide the development of an intervention programme appropriate for use in South Africans with late-stage OA (Edries, Jelsma & Maart 2013; Parker, Jelsma & Stein 2014). The information was categorised according to the intervention components: education, exercise and relaxation. Further categories explored were: setting or site and length of delivery. The field investigation located sites that would be appropriate for running such an intervention and which could be used in a study to investigate the effectiveness of the intervention. The field investigation was conducted through site visits, where geographical location, patient group, safety, accessibility and physiotherapy availability were considered. Sites were considered if they had adequate facilities (a hall or gym space, chairs and were private), were accessible via public transport and provided treatment for patients with late-stage OA. Patients were diagnosed as having late-stage OA radiographically by the orthopaedic consultants at the healthcare facilities, as classified according to the Kellgren and Lawrence plain radiograph classification scale (Kijowski *et al*. 2006).

## Results

A 6-week, physiotherapist-led exercise and education intervention was designed based on the literature reviewed and considering the South African health-care context within the ICF framework (Brady *et al*. 2003; Edries *et al*. 2013; Gatchel *et al*. 2014; Hurley *et al*. 2007a; Hurley & Walsh 2009; Jessep *et al*. 2009; Parker 2013). While health education is a cornerstone to improving knowledge, self-efficacy and health promotion, it does not necessarily lead to behaviour change and an empowered patient (Parker, Jelsma & Stein 2014). Parker *et al*. (2014) argue that ‘in order for changes in knowledge and belief to translate into behaviour changes, cognitive behavioural approaches with goal setting need to be adopted’ (2014:14). Therefore, a combined educational approach, with a strong focus on the physical aspects of exercise in particular was adopted for the intervention in order to improve function and manage the disability associated with OA. The specific information, which was drawn from the literature and used to develop the intervention, will be presented first, followed by the findings from the site inspections (Brady *et al*. 2003; Edries *et al*. 2013; Gatchel *et al*. 2014; Hurley *et al*. 2007a; Hurley & Walsh 2009; Jessep *et al*. 2009; Parker 2013).

## Theoretical Model

The intervention used the principles of social learning and cognitive behavioural theory, such as problem solving and goal setting, which aim to achieve behaviour change and build confidence, as its primary theoretical model (Lorig & Holman 2003). Instructive health education programmes have shown limited effectiveness in managing function in people with chronic diseases (Von Korff *et al*. 2005). Therefore, in order for knowledge and beliefs to translate into behaviour changes with positive impact on function and quality of life, cognitive behavioural approaches; primarily through goal setting, need to be adopted.

## Educational Content

Despite education alone not leading to desirable behaviour change, studies have been seen to support the use of education to reduce pain and anxiety in patients awaiting total hip arthroplasty (Von Korff *et al*. 2005). In order for education to be effective, the components need to be relevant to the management of OA, focusing on self-management in terms of psychological wellbeing, quality of life and self-efficacy (Ottawa Panel 2011).

An example of a commonly used patient education intervention is the Chronic Disease Self-Management Program (CDSMP) (Stanford School of Medicine 2015). The CDPSMP is a workshop given over two-and-a-half hours, once a week, for 6 weeks, in a community setting. Educational topics covered include: (1) techniques to deal with problems such as frustration, fatigue, pain and isolation; (2) appropriate exercise for maintaining and improving strength, flexibility and endurance; (3) appropriate use of medications; (4) communicating effectively with family, friends and health professionals; (5) nutrition; (6) decision making; and (7) how to evaluate new treatments. Each participant in the workshop receives a copy of the companion book, ‘*Living a Healthy Life with Chronic Conditions’* and an audio relaxation CD, ‘*Relaxation for Mind and Body’.* ‘Classes are participative, where mutual support and success build the participants’ confidence in their ability to manage their health and maintain active and fulfilling lives’ (Stanford School of Medicine 2015). Lorig *et al*. (2001) found that the CDSMP produced significant improvements in health distress and reductions in ambulatory healthcare for chronic illnesses including unspecified arthritis (Lorig *et al*. 2001).

The Ottawa Panel (2011) has recently reviewed evidence to develop practice guidelines for patient education programmes in the management of OA (Ottawa Panel 2011). They recommended that a self-management programme should apply social learning theory to empower participants moving them away from the traditional passive information-receiving role. When interventions contain self-management education based on social learning theory, improvements are recorded in pain, disability and overall health status (Wu *et al*. 2011). The model of education used is an important aspect to consider thereby avoiding the pitfalls of depending on an individual therapeutic relationship to achieve a treatment effect.

For the development of the intervention for this study, the educational topics were therefore incorporated into a progressive goal-oriented programme allowing the participants to implement self-management skills based on knowledge. Each week focused on a specific topic identified as relevant and useful for patients through the literature review (Table 1). In addition, the educational topics were incorporated into a workbook titled: ‘Living with Osteoarthritis’ covering in detail the same topics discussed in the weekly sessions. Both the workbook and the educational sessions of the intervention programme aimed at facilitating the development of the core self-management skills of problem solving and decision making. To allow for a transfer of knowledge into action, the handbook included an exercise diary, SMART (specific, measurable, attainable, realistic and timed) goal setting sheets and problem-solving tasks (Parker *et al*. 2014).

**TABLE 1:** Topics and content of the ‘Living with Osteoarthritis’ workbook.

Topic for the week	Content
Week 1: Osteoarthritis, self-management and exercise	What is osteoarthritis Management options Exercise dos and don’ts Types of exercise Steps to success with exercise Exercise routines
Week 2: Managing common symptoms	What is pain? Flare-ups of pain and what can I do? Stiffness and how to manage Activity limitations because of symptoms Assistive devices Pacing Fatigue Depression, frustration, isolation
Week 3: Stress management	What is stress? Managing stress Communication with your family Sleep routine Planning ahead Relaxation techniques
Week 4: Eating well	Two schools of thought: choosing a diet The role of food groups Eating guidelines Losing weight Steps to healthier eating
Week 5: Medication and disease-related problem solving	Analgesic drugs Anti-inflammatories Anti-spasmodic drugs Epileptic and anti-depressants The analgesic ladder Making informed decisions
Week 6: Continuing as a successful self-manger	Action planning for the future Reflection on changes

*Source*: http://open.uct.ac.za/handle/11427/12697

The workbook was divided into six chapters, encouraging participants to work through the subjects at regular intervals simultaneously with the subjects taught in contact sessions. As self-management, in particular with exercise components, has been found to have an overall positive reduction of pain, the workbook also included information on exercise, exercise programmes and planning of such (Ottawa Panel 2011).

## Skills Development

A participation agreement was designed to be signed by all participants. The agreement aimed at facilitating open discussion while maintaining respect and confidentiality among the group members. The agreement also aimed at encouraging commitment to the programme and facilitating active participation in skills development. Further skills development included the workbook section of ‘activity planning’. Here the participants were taught the skills of developing action plans in accordance with content learned at the workshop. This was designed to develop the skill of progressively advancing goals set on a weekly basis.

## Exercise Content

OARSI and the National Institute for Health and Care Excellence (NICE) guidelines state that local muscle strengthening exercises and general aerobic fitness should be part of core treatments for people with OA, irrespective of age, co-morbidity, pain severity or disability (McAlindon *et al*. 2014; O’Mahony & Oliver 2008). These exercise recommendations are based on evidence with large effect sizes. Positive effects in terms of pain reduction and improvements in function have been reported after a combined approach of aerobic and strengthening exercise (Escalante *et al*. 2010; Hiyama *et al*. 2012; van Tulder *et al*. 2000). One such programme was the previously mentioned ESCAPE knee pain programme, which combined strengthening and aerobic exercises in a progressive manner.

The exercise components for the intervention designed for the South African population combined aerobic, strengthening and flexibility activities and was largely based on the ESCAPE knee pain programme by Hurley *et al*. (2007a, 2009) (Table 2). The content of the programme falls within the parameters of the OARSI and NICE guidelines (McAlindon *et al*. 2014; O’Mahony & Oliver 2008). Furthermore, the ESCAPE knee programme is similar to other evidence-based exercise programmes that have been used in South African populations with chronic pain (Parker *et al*. 2009, 2014). The exercise programme is designed to be applied in a group setting and is easy to modify for the individual participant depending on their current state of symptoms and abilities. Exercises were chosen on a basis of safety, simplicity (no equipment needed) and ability to adapt to progression in aerobic fitness and muscle strength. The exercise programme was designed to start at 20 minutes in length during the first week with the duration increasing by 10% each week. Instructions for the exercises included in the workbook indicated that all aerobic exercises should be carried out at 60% of maximum heart rate based on the Borg scale of exertion using the descriptive term ‘somewhat hard’ (Borg 1998). Flexibility and strengthening exercises focused on muscle groups relevant to the participants with diagnoses of OA of the lower limbs.

**TABLE 2:** Exercises included in the intervention.

Type of exercise	Aim	Activities	Progression
Walking	Warm up Improve Cardiovascular fitness	Walking on the spot	Increase speed and reps, incorporate arm swings and boxing. Take bigger steps. Lift knees higher
Quads and hamstring strength	Isotonic lower limb strength	Knee extension exercises. Kicking backwards towards buttocks	Increase speed and reps
Upper limb movements	Range of movement improvement for upper limbs and trunk	Reaching up and sideways, while walking on the spot	Increase speed and coordination with different movements
Standing on one leg	Balance and coordination	Single lag stance, support wall	Increase reps, stand on matt (soft), no support wall
Sit to stand	Strength, control, function	With arms folded sit to stand from chair or bench	Decrease height, increase time
Floor squats	Strength and control	Standing near support, squatting as far as possible	Increase time. Exercise only introduced in advanced stages
Stretches	Range of motion and flexibility	Stretches of major upper limb and lower limb muscle groups	Not applicable
Interactive activities	Social, fun, CV, balance, concentration, coordination	Large gym-ball bouncing between participants, stepping and saying each other’s names, etc.	Use smaller ball and throw to each other, add more difficult games, stand in line and pass ball backwards

*Source*: Authors’ own work

## Relaxation Content

Late-stage OA predominantly presents as chronic pain with several psychosocial factors influenced by/influencing the pain experience (Gatchel *et al*. 2014). Therefore, it is appropriate to consider including other non-pharmacological management approaches in addition to education and exercise. As discussed, the educational component is based on a cognitive behavioural approach, a model shown to have a positive effect in the treatment of people with chronic pain (Gatchel *et al*. 2014). Cognitive behavioural interventions promote significant improvements in multiple psychosocial dimensions of chronic pain (e.g. coping, pain behaviour and social functioning) (Lunde & Nordhus 2009) and usually include a component of relaxation or mindfulness training (Gatchel & Rollings 2008; McCracken & Turk 2002).

Diezeman (2011) reports that progressive muscle relaxation (which entails a short contraction of a muscle group followed by complete relaxation of the same group) is the most commonly used and best studied relaxation method used by physiotherapists. The technique leads to distraction from pain, allowing internal focus of control and improvement of self-efficacy (Diezeman 2011). Targets of relaxation training are ‘Improvement of body awareness and stress management, shielding from sensory stimuli as well as serving as a sleeping aid’ (Diezeman 2011). This method has good compliance with patients because it is easy to learn (Diezeman 2011).

While the above evidence for relaxation training is mainly based on populations with chronic low back pain, headaches and fibromyalgia, recent literature highlighting the central nervous system changes present in late-stage OA supports the application of the approaches in this population (Lluch *et al*. 2014). The relaxation component of the intervention programme was designed based on the literature available (Diezeman 2011; Gatchel & Rollings 2008; Lluch *et al*. 2014; McCracken & Turk 2002). The relaxation component includes appropriate imagined visualisations/relaxation journeys combined with contract/release of major muscle groups. The relaxation component was designed to be conducted with the participants sitting or lying down on comfortable mats with pillows for support at every session. The environment was quiet and shielded from outside noise and distraction. In addition to the relaxation session conducted at the weekly workshop, a CD was recorded for participants to use in their home environments.

## The Sites

Social isolation is a known problem for patients with chronic illnesses (Chan & Chan 2011) and there are positives to be derived from group treatments, in terms of social interaction. The intervention was therefore designed for a group setting of a maximum of 12 participants. This number of participants allows for individual attention, as well as the group benefits of social learning, that is, motivation from seeing other participants achieving goals benefitting their health and vicarious learning (Chan & Chan). Further to this, the sites selected for delivery of the intervention needed to be familiar to participants, accessible and safe. Sites at public health institutions were identified, which had appropriate space, privacy and transport availability.

## Physiotherapist-led Facilitation

One of the biggest obstacles in modern healthcare practice is adherence to guidelines and despite evidence-founded recommendations, their practice is often not followed (Walsh & Hurley 2009). However, the OARSI guidelines do recommend exercises be prescribed by an appropriate specialist (McAlindon *et al*. 2014). Reliance on general practitioners to prescribe exercises may be inappropriate both from a time and skill perspective, whereas physiotherapists, who are skilled in assessment, exercise prescription, biomechanical dysfunction and behavioural interventions (pacing and action planning), are ideally placed to provide this specialist service (McAlindon *et al*. 2014; O’Mahony & Oliver 2008).

## Length of Delivery

CDSMPs commonly range from 6–10 weeks in length (Foster *et al*. 2007; Marks, Allegrante & Lorig 2005; Parker *et al*. 2014). Interventions lasting for longer periods of time have greater dropout rates, which may negatively affect outcome. The Stanford Education Research Centre has tested several chronic disease management programmes and has found that an appropriate length of delivery to be between 6 and 7 weeks. This allows for enough time to accommodate behaviour changes, but at the same time is not regarded as excessively long by participants (Lorig *et al*. 2005b; Lorig & Holman 2003). Therefore, for the intervention designed for a South African population with late-stage OA where challenges are faced in terms of adherence, a 6-week period was selected.

## Conclusion

This paper illustrates the process followed to develop an evidence-based 6-week physiotherapist-led exercise and intervention for South African patients with late-stage OA of the hip or knee. The ‘Living with Osteoarthritis’ bio-psychosocial intervention was specifically developed for the management of pain, function, disability and health-related quality of life in patients waiting for arthroplasty of the hip or knee because of OA. The workbook can be accessed at: https://open.uct.ac.za/handle/11427/12697. The effectiveness of the intervention has been tested in a randomised trial, the results of which are reported elsewhere.
